# Ten Steps in Adoption of Emerging Procedural Techniques: An Experience With Ethanol Ablation of Vein of Marshall (VOM)

**DOI:** 10.7759/cureus.24948

**Published:** 2022-05-12

**Authors:** Talha Wahid, Hafiza Khan, Mustafa M Dohadwala

**Affiliations:** 1 Research, Baylor Scott & White The Heart Hospital, Plano, USA; 2 Cardiac Electrophysiology, Baylor Scott & White The Heart Hospital, Plano, USA

**Keywords:** procedure training, teaching procedures, mitral annular flutter, atrial fibrillation, ethanol ablation, vein of marshall

## Abstract

Proceduralists must update their skill sets to provide patients with better care because of the addition of new and effective strategies post-training. For example, the current procedural strategy of pulmonary vein isolation for treating persistent atrial fibrillation (AF) is inadequate. However, the addition of ethanol ablation of the vein of Marshall (VOM), a relatively new procedural technique, can improve outcomes. Furthermore, the purpose of this report was to briefly explain VOM ethanol ablation, its role in atrial fibrillation and atypical flutter ablation, and to provide a template for performing a new procedural technique in the field.

## Introduction

Over the last decade, surgical ablations have become the gold standard for any drug treatment for recurrences of atrial fibrillation and quality of life. However, the current standard of care for persistent atrial fibrillation (AF) using catheter ablation has limitations [1]. Except for the strategic procedure of pulmonary vein isolation, expanding lesion sets to include linear lesions or ablate areas of irregular electrical activity has been inconsistent in improving outcomes [2]. Furthermore, recurrent AF and atrial tachycardias lead to repeat ablation procedures. One emerging technique, ethanol ablation of the vein of Marshall (VOM), provides an effective strategy to treat patients with AF. 

The VOM, an embryological remnant of the left superior vena cava, has been suggested as a source of AF incitement because of parasympathetic and sympathetic innervation that affects the electrophysiology of atrial tissue [3]. In addition, VOM is located within the mitral isthmus, which is important for perimitral atrial tachycardia. As a result, ablation by retrograde ethanol infusion is a possible technique to eliminate AF triggers and VOM innervation [4]. In a recent study published in the Circulation: Arrhythmia and Electrophysiology Journal, 88.9% of 713 patients had successfully underwent VOM of ethanol infusion and a total of 14 patients experienced serious complications [5]. In other words, this technique is highly feasible and has a low rate of serious complications. 

Because of emerging and effective techniques, physicians must update their skill sets to provide patients with state-of-the-art procedures that were not present during training. Using ethanol ablation of the VOM as an example, we suggest a 10-step template for any physician to successfully adopt any emerging procedure. 

## Technical report

Step 1 - decide whether the data is sound and whether patients would benefit from the procedure. Is it safe, effective, and applicable to your current patient population?

For example, there is a growing compendium of data to confirm the role of ethanol ablation of VOM for mitral flutter and persistent atrial fibrillation. By studying the data, we properly weighed the benefits and harm. Although AF ablation success rates are increasing and complication rates are decreasing, there remains room for improvement. Opinions diverge regarding additional lesion sets once pulmonary vein isolation is completed, including mitral line, roof line, floor line, coronary sinus ablation, etc. A mitral line achieving bidirectional block can be challenging to perform due to epicardial connections via the coronary sinus/great cardiac vein and VOM.

Step 2 - have an honest assessment as to whether you have the skill set to implement this new technique

Electrophysiologists are familiar with percutaneous sheath management, contrast injection, and wire manipulation, which are all requisite skills. But, interventional cardiologists, compared to cardiac electrophysiologists, are more familiar with ethanol injection and balloon angioplasty. With supplemental education (see Step 3) and support from our cardiac interventional team, as a heart team, we determined this procedure fit into the cardiac electrophysiologist’s skill set and could be performed safely.

Step 3 - find your knowledge gaps and fill them

Prior to scheduling the first patient procedure, we reviewed the angioplasty using interventional cardiology textbooks. We also studied articles regarding VOM anatomy and ethanol ablation pathophysiology and procedural steps. Most importantly, we reviewed complications regarding ethanol injection, such as complete heart block or ventricular tachycardia from scar. Although some of the complications arise due to anatomical reasons, we learned how to adjust our technique and reduce the risk of anaphylaxis.

Step 4 - prepare yourself and your team. Confirm you have “buy-in” from your colleagues, lab staff, and anesthesiologists

The authors and entire staff were then given a comprehensive supply list and step-by-step “how-to" for the procedure prior to scheduling a patient for the VOM ablation. The supply list included a 5 Fr left internal mammary artery (LIMA) catheter, large-curved 8.5 Fr steerable sheath (Abbott Agilis, Biosense Webster Vizigo), 8.5 Fr SL-1, over-the-wire 1.5 mm to 2.5 mm x 6 mm to 10 mm angioplasty balloons (Medtronic Sprinter Semicompliant), balloon insufflator, 014 coronary wires, y-shaped hemostatic valve (i.e., Abbott Copilot, Teleflex Guardian), iodinated contrast, and dehydrated ethanol (10 cc).

Step 5 - choose the right patient! Successful and safe completion of the first procedure is key to ongoing success, so choose wisely

During the consent process, patients were informed that this is a newer yet promising technique based on the known data. They were made aware that the major risk was perforation leading to pericardial tamponade, but this risk was not substantially higher than that seen during conventional ablation. Also, there was a possibility the VOM was not suitable for ethanol injection. 

Our first patient was ideal for VOM ethanol injection as he had prior complex AF ablation that included pulmonary vein isolation, posterior wall isolation, and a mitral line that did not achieve block. Subsequently, we selected those with confirmed mitral flutter and have expanded therapy to patients with first-time ablations for long-standing persistent atrial fibrillation and for repeat ablations despite durable pulmonary vein isolation.

Step 6 - set up your “team.” Preplanning with all the necessary equipment, staff, and contingency plans are the keys to successfully implementing a new technique!

We assembled an experienced team that would adapt to this new procedure. Cardiac anesthesiologists facile in the EP laboratory were chosen. Given skills in percutaneous and peripheral arterial interventions, EP technologists and nurses who had cardiac catheterization lab experience were selected. Prior to performing the procedure, we performed a mock VOM ethanol injection in preparation for our first live patient. Importantly, an interventional cardiologist co-scrubbed the first three VOM ethanol injection cases to help guide balloon size, wire selection, balloon insufflation pressure, etc.

Step 7 - perform the procedure

**Figure 1:**
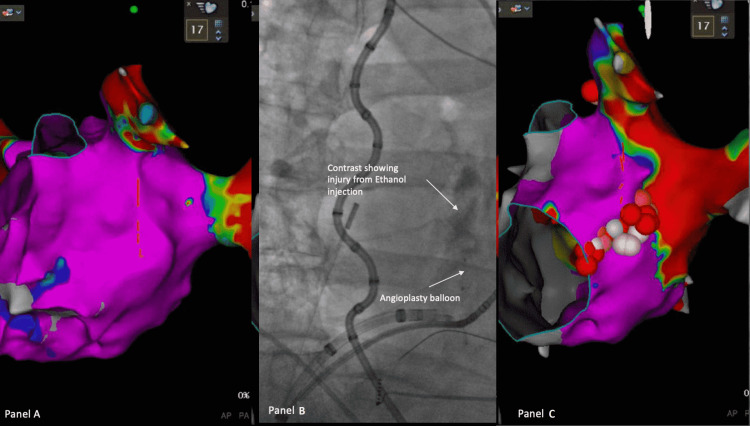
Left atrial mapping and fluoroscopy of ethanol injection in the vein of Marshall. (A) A voltage map of lateral view of left atrium prior to pulmonary vein isolation and ethanol ablation of vein of Marshall. (B) The contrast that has been injected through the angioplasty balloon after ethanol ablation confirms venule injury. (C) A voltage map of lateral view of left atrium after ethanol ablation of vein of Marshall, pulmonary vein isolation, and endocardial ablation of mitral annulus. The large area of scar (red) along the coumadin ridge and inferior to the left lower pulmonary vein is due to ethanol ablation of the vein of Marshall.

The left atrium was first mapped (Figure 1, panel A) with a 5 Fr LIMA catheter, balloon, angioplasty wire, manifold, and insufflator. The attending electrophysiologist and first assistant worked together. While the electrophysiologists would torque the LIMA catheter and advance the balloon in position, the assistant would pin the wire and help inject contrast and ethanol. A total of three injections of 3 cc each of dehydrated ethanol were injected over one minute each. The contrast was injected between injections to assess balloon position and integrity of the vessel (Figure 1, panel B). Endocardial and coronary sinus ablation was typically required to achieve block across the mitral line, which was confirmed with differential pacing (Figure 1, panel C). 

Step 8 - debrief and rebrief. What went well? What can we do better? All members of the team should be involved

We debriefed as a team to improve subsequently, discussing the size of balloon, level of insufflation, wire selection, etc. With review of angiography, the interventionalists were instrumental in training the authors’ eye to balloon size for subsequent cases. Also, balloon insufflation was the safest at lower pressures (3-4 mm Hg) to avoid dissection of the delicate VOM. When 5 Fr LIMA was not available, we found the 6 Fr LIMA more challenging to maneuver for the VOM. Also, we have been using a long 8.5 Fr SL-1 to place our decapolar coronary sinus catheter. If the ablation catheter does not easily go into the coronary sinus to complete mitral block, we advance the SL-1 over the more pliable decapolar catheter and then replace the decapolar catheter with the ablation catheter. In one case, we had localized staining with contrast injection after ethanol injection and were reassured by a study confirming this feature did not have an adverse procedural outcome.

Step 9 - collect your data

Since the first procedure, we have been collecting our own procedural data to compare our results to other centers and to better understand when our techniques and patient selection benefit from adjustments. For instance, we are confirming the presence of mitral block after VOM ethanol ablation and how often endocardial mitral annular and/or coronary sinus radiofrequency ablation is required for block. 

In total, we have successfully performed VOM ethanol in 14 of 15 (93%) attempted procedures between April 2021 and April 2022. Two of the 15 patients were first-time ablations while the remainder were redo ablations. The strategy entailed re-isolating pulmonary veins if needed, performing roof line or posterior wall isolation, and achieving mitral block with ethanol injection and endocardial mitral isthmus/coronary sinus ablation. In the 14 patients with successful injection of ethanol, mitral block was achieved in 13 of 14 (93%) patients when combined with endocardial ablation of the mitral isthmus and coronary sinus ablation. Mitral block was not achieved in the one patient due to the inability to navigate the ablation catheter into the coronary sinus. There has also been no change in recovery time and patients routinely go home the same day.

Of the 14 patients with VOM ethanol injection, there has been an 86% freedom from AT/AF with two patients with recurrences (14%) over a median time frame of 235.5 days. One of the recurrences required repeat ablation, targeting fractionated activity in the septum and coronary sinus. The other was treated with cardioversion and antiarrhythmic therapy and has done well in the interim. There have been no pericardial effusions or other complications. The one failed ethanol injection was due to a localized dissection of the VOM. Of the one patient where ethanol injection was not successful, inferolateral mitral line was performed along with coronary sinus ablation and mitral block was successfully achieved. This patient has had no recurrence. At this time, seven of the 15 (47%) patients remain on antiarrhythmic therapy. Although our data collection is at an early stage, previous studies have indicated the impact of this procedure on patients. 

Step 10 - spread the word! Once you have successfully implanted this new procedural technique, let your colleagues know that this is available to their patients 

In the ever-changing and evolving fields of medicine, there are incremental gains found frequently during procedures. In our mind, VOM ethanol ablation fits in the less frequently encountered “game-changer” category. We are excited to share our relatively new journey in performing this procedure and believe others will share our enthusiasm.

## Discussion

Out of the 15 patients that underwent VOM ethanol ablation, we successfully performed VOM ethanol in 14 patients (93%) between April 2021 and April 2022. And of the 14 patients with successful injection of ethanol, mitral block was achieved in 13 patients (93%) when combined with endocardial ablation of the mitral isthmus and coronary sinus ablation. The high success rate of our procedural strategy may be due to re-isolating pulmonary veins when needed, performing roof line or posterior wall isolation, and achieving mitral block with ethanol injection and endocardial mitral isthmus/coronary sinus ablation. Even though mitral block was not achieved in one patient and two patients had recurrences, our data is comparable with the results of other studies.

In 2009, Valderrabano et al. performed vein of Marshall (VOM) ethanol ablation in canines demonstrating local ablation, regional denervation effects, and decrease in effective refractory period [6]. In 2012, the same group demonstrated the VOM consistently lied in the reentrant circuit of perimitral flutter demonstrated by entrainment [7]. In the VENUS trial, Valderrabano et al. enrolled patients with persistent AF undergoing their first catheter ablation procedure to radiofrequency ablation alone (pulmonary vein isolation plus additional lesions per treating physician’s discretion) compared to radiofrequency ablation and VOM ethanol ablation. The freedom from AF/AT was 49% in the ablation+VOM group and 38% in the ablation-only group (p = 0.04) [8]. Derval et al. followed 75 consecutive persistent AF patients with VOM ethanol ablation, pulmonary vein isolation, mitral line, roof line, and caval-tricuspid valve ablation. In this cohort, 72% were free from arrhythmia at 12 months [9]. 

Although the procedure is largely successful, complications do arise. When ethanol is injected into septal perforators for septal reduction of hypertrophic cardiomyopathy, the complications are largely specific to the septal coronary anatomy, such as complete heart block or ventricular tachycardia from scarring. In about 10-15% of patients, the VOM is not amenable to successful ethanol ablation due to anatomy [8,5]. Contrary to ethanol leaking into the left anterior descending artery, ethanol leakage into the right atrium dilutes and should cause no untoward effect. Most significantly, manipulation of the tools and ethanol injection into the VOM can lead to perforation with tamponade and dissection of the vessel [10]. Although complications can arise, the likelihood of tamponade is not much greater than with conventional ablation [5]. And rare complications, such as anaphylaxis, can be reduced by injecting ethanol slowly [11]. 

Furthermore, the benefits of this procedure outweigh the risks. For example, in 713 consecutive patients, Kamakura et al. demonstrated the feasibility of VOM ethanol infusion in 88.9% of cases. When successful, the mitral line block achieved 95.8%. There was a 2% complication rate, with seven tamponades, four strokes, one anaphylactic shock, one AV block, and one appendage [5]. In a meta-analysis of four studies with 804 AF patients, the patients treated with VOM ethanol ablation had lower risk of recurrent AF/AT without an increase in complications compared to patients treated with conventional ablation [12].

## Conclusions

Ethanol ablation of the vein of Marshall (VOM), a relatively new procedural technique, can decrease the recurrence of arrhythmias in patients. The addition of this technique can improve upon the common practice of pulmonary vein isolation. Moreover, the ethanol ablation of the vein of Marshall (VOM) is one example of emerging techniques. With a methodical approach to navigate procedures, the quality of emerging techniques can grow efficiently. We believe this 10-step approach can be generalized to the ever-evolving cardiovascular space, where new technologies emerge quickly. This step-by-step process can help physicians update their skill sets and provide patients with state-of-the-art procedures that were not present during training.
